# Comparing Efficacy and Safety of Empirical vs. Guided Therapy for Non-cardiac Chest Pain: A Pragmatic Randomized Trial

**DOI:** 10.3389/fmed.2021.605647

**Published:** 2021-02-15

**Authors:** Noor Purdah Abdul Kadir, Zheng Feei Ma, Muhammad Ilham Abdul Hafidz, Chandramouli Annamalai, Thevaraajan Jayaraman, Nurhazwani Hamid, Siti Norhasliza, Azliani Abd Aziz, Zurkurnai Yusof, Hady Lee, Yeong Yeh Lee

**Affiliations:** ^1^School of Medical Sciences, Universiti Sains Malaysia, Kota Bharu, Malaysia; ^2^Gastroenterology Unit, Faculty of Medicine, Universiti Teknologi MARA, Shah Alam, Malaysia; ^3^Gut Research Group, Faculty of Medicine, Universiti Kebangsaan Malaysia, Kuala Lumpur, Malaysia

**Keywords:** non-cardiac chest pain, GERD (gastroesophageal reflux disease), quality of life, dexlansoprazole, theophylline

## Abstract

**Background:** Non-cardiac chest pain is common with two-thirds due to gastroesophageal reflux disease (GERD).

**Objective:** To evaluate the effectiveness and safety of guided vs. empirical therapy in non-cardiac chest pain.

**Methods:** Adults with normal angiogram or stress test were randomized into either a guided or empirical group. In the guided group, after the ambulatory pH-impedance test, if GERD then dexlansoprazole 30 mg/day for 8 weeks, but if functional or hypersensitive chest pain, then theophylline SR 250 mg/day for 4 weeks. In the empirical group, dexlansoprazole 60 mg/day was given for 2 weeks. The primary outcome was global chest pain visual analog score (VAS) and secondary outcomes were Quality of Life in Reflux and Dyspepsia (QOLRAD), GERD questionnaire (GERDQ), and pH parameters, all determined at baseline, 2nd and 8th weeks.

**Results:** Of 200 screened patients, 132 were excluded, and of 68 randomized per-protocol, 33 were in the guided group and 35 in the empirical group. For between-group analysis, mean global pain scores were better with guided vs. empirical group at 8th week (*P* = 0.005) but not GERDQ or QOLRAD or any of pH measures (all *P* > 0.05). For within-group analysis, mean QOLRAD improved earliest at 8th week vs. baseline (*P* = 0.006) in the guided group and 2nd week vs. baseline (*P* = 0.011) in the empirical group but no differences were seen in other secondary outcomes (*P* > 0.05). No serious adverse events were reported.

**Conclusions:** Guided approach may be preferred over short-term empirical therapy in symptom response, however QOLRAD, acid-related symptoms, or pH measures are not significantly different (trial registration ID no. NCT03319121).

## Introduction

Non-cardiac chest pain is defined as recurrent episodes of chest pain, identical to ischemic heart pain but in the absence of a cardiac cause (1, 2). Patients are often associated with poorer quality of life (QOL) and healthcare costs were expensive (3). The disorder is common but global epidemiology especially of Asia is relatively limited (4, 5). The average annual prevalence based on six population-based studies was estimated at 25% (2).

While non-cardiac chest pain may be attributed to gastrointestinal, musculoskeletal, pulmonary, and psychological causes but almost two-thirds is related to gastroesophageal reflux disease (GERD) (6). In real-life practice, the proton-pump inhibitor (PPI) test, where PPI is given for 2 weeks, is the typical empirical approach, and this is effective in more than half of patients (7–9). However, about a third or more of patients may remain symptomatic because of a different diagnosis, and the patients would eventually end up having the pH or pH-impedance test thus significantly adding up the treatment costs.

If not the empirical PPI test, the other approach would be therapy guided by the results of a 24-h pH or pH-impedance test, at the onset (10). As the gold standard, ambulatory pH or pH-impedance test can phenotype true GERD from hypersensitivity or functional chest pain (11). With the guided approach, if the pH test indicates true GERD, then PPI would be given for a longer duration beyond 2 weeks. However, if the pH test indicates otherwise, then the effective treatment for non-acid pain is likely a pain modulator which may include a theophylline (12), a tricyclic antidepressant (e.g., imipramine) (13), selective serotonin reuptake inhibitor or SSRI (e.g., sertraline) (14), or serotonin noradrenaline reuptake inhibitor or SNRI (e.g., venlafaxine) (15) for a duration typically ranging between 4 and 12 weeks (16).

It is unknown what would be the next recommended approach i.e., empirical or guided once cardiac causes of chest pain are excluded. Therefore, our study aimed to evaluate the effectiveness and safety of empirical therapy vs. guided therapy in unexplained non-cardiac chest pain.

## Methods

### Study Design and Eligibility

This was a prospective, single-center, open-label, pragmatic, randomized clinical trial involving consecutive participants with unexplained chest pain. Participants were patients recruited from outpatient and gastroenterology clinics of Hospital Universiti Sains Malaysia (USM), a tertiary University hospital situated in northeastern Peninsular Malaysia. All participants provided signed informed consent before study enrolment. Inclusion criteria were patients aged 18–80 years with chest pain but normal angiogram or a negative stress test or a normal electrocardiogram and cardiac enzymes or a normal CT coronary angiography. Exclusion criteria included recent use of any medications (including nitrates, calcium channel blockers, histamine receptor blockers, and PPI) that might affect the upper gastrointestinal (GI) tract, previous surgeries of the upper GI tract, presence of peptic ulcer disease, and upper GI tract malignancies found during the endoscopy and the presence of major motility disorders. Patients with chronic, debilitating, or life-threatening medical conditions and the presence of overt psychiatric or moderate to severe psychological disturbances (including anxiety and depression) were also excluded.

The study was approved by the Human Research Ethics Committee, USM (reference: USM/JEPeM/14070265) and registered with the ClinicalTrials.gov (trial registration ID no. NCT03319121). The study results were reported following the Consolidated Standards of Reporting Trials (CONSORT) guidelines and the World Medical Association Declaration of Helsinki.

### Study Procedures

During screening, participants were asked to complete questionnaires including the Malay translated and validated versions of the gastroesophageal reflux disease questionnaire (GERDQ), Quality of life in Reflux and Dyspepsia (QOLRAD) (17), and current visual analog score (VAS) of their chest pain. The chest pain VAS was a global symptom score from 1 to 10 elicited from participants by taking a combined account of frequency, intensity, and duration of pain with 0=no symptom and 10=maximal symptom. Besides, weight (kg), height (m), and body mass index (kg/m^2^) of participants were measured. All participants consented for an upper endoscopy, high-resolution esophageal impedance manometry, and ambulatory 24-h pH-impedance test (only performed in the guided therapy group).

### Upper Endoscopy

Endoscopy (Model GIF-140 and GIF-160; Olympus Medical Systems, Tokyo, Japan) was performed by a single endoscopist (YYL). When present, the degree of erosive esophagitis was documented based on the Los Angeles (LA) classification (18). Biopsies were taken for urease test and/or histology to determine the presence of *Helicobacter pylori* infection. Patients diagnosed with *Helicobacter pylori*-associated dyspepsia, peptic ulcer disease, and upper GI tract malignancies were excluded and managed accordingly.

### High-Resolution Esophageal Impedance Manometry

A solid-state probe (Laborie Medical Technologies, Mississauga, Canada) that consists of 36 pressure channels and eight impedance sensors was placed across the esophagus and upper stomach of participants. The catheter was inserted nasally after lignocaine spray in the sitting position. After rest, participants were given 10.5-mL water swallows. Upon completion of all water swallows, the probe was removed. The upper border of the lower esophageal sphincter (LES) was measured during the rest period, and participants with major motility disorders especially achalasia were excluded from the study and managed accordingly.

### Ambulatory 24-hour pH-Impedance Monitoring

The ZepHr® pH-impedance probe (Diversatek Healthcare, Highland ranch, USA) consists of one pH sensor located 5 cm from the tip of the catheter, and six impedance sensors spaced regularly above the pH sensor. Before insertion, the probe was calibrated with buffers at pH 4.0 and 7.0. After lignocaine spray, the catheter was passed nasally, typically in a sitting position, and the pH sensor placed 5 cm above the upper border of LES. The recording was started when the probe was placed in its correct location. Participants were instructed to record any events in a given diary. After 24-h, the probe was removed and reflux events analyzed subsequently.

### Study Intervention

Using free online software (http://stattrek.com/statistics/random-number-generator.aspx), a random table was generated with the guided group coded as number 1 and the empirical group coded as number 2. Simple randomization of participants into groups was performed in a consecutive manner based on the random table generated.

In the empirical therapy group, participants were given 60 mg dexlansoprazole (Dexilant®, Takeda Pharmaceuticals, Japan) daily for two (2) weeks. In the guided therapy group, ambulatory pH-impedance test was performed first, and subsequent therapy was guided based from results of the test. Based on results of the pH-impedance test, two groups of participants (i.e., group 1: GERD and group 2: functional chest pain or hypersensitive esophagus) were identified. Participants from group 1 with GERD (defined as % total acid exposure time >6% with positive symptom association >95%) were treated with dexlansoprazole 30 mg daily for eight (8) weeks. For those in group 2 with functional chest pain (defined as % total acid exposure time <6% with negative symptom association <95%) or reflux hypersensitivity (defined as % total acid exposure time <6% with positive symptom association >95%), they were treated with theophylline SR 250 mg daily for four (4) weeks.

At the end of the second (2nd) and eight (8th) week of study intervention, all participants were asked to complete the VAS, GERDQ, and QOLRAD questionnaires. Only participants in the guided therapy group were offered to repeat the ambulatory 24-h pH impedance test at the end of the 2nd week. Adverse events were also documented at each visit. At the same time, their drug compliance was checked by pill-counting during planned hospital visits. Participants were considered as “drop-out” if they missed their medications for more than 50% of the time or if they were lost to follow-up.

### Data and Statistical Analysis

Based on our clinical observation and exploratory pilot data, the response within each group was normally distributed with a standard deviation of 0.8. When the true difference in the experimental and control means was 0.6, each randomized group would require 29 participants with a probability (power) of 0.8. Assuming that the drop-out rate was anticipated at 20%, 35 participants were needed in each group.

The primary outcome was symptom improvement based on differences in the mean chest pain VAS at 2nd and 8th weeks from baseline. Secondary outcome included differences in quality of life (QOLRAD), GERDQ scores at 2nd, and 8th weeks from baseline, and also reports of adverse events. Additional secondary outcomes were within-group differences in the pH parameters (mean 24-h pH, % time acid exposure, and DeMeester score) in the guided group. Both per-protocol and intention-to-treat analyses were performed in the current study. Data were analyzed using SPSS version 21.0 software (SPSS Inc., Chicago, IL, USA). All data were reported as mean ± standard deviation unless mentioned otherwise. Comparison between groups (i.e., guided vs. empirical) and within groups (i.e. 2nd vs. 8th week) was made using *t*-test and repeated measures analysis of variance (ANOVA) with Bonferroni adjustment. All tests were considered significant at *P* < 0.05.

## Results

### Participant Characteristics

**Figure 1:**
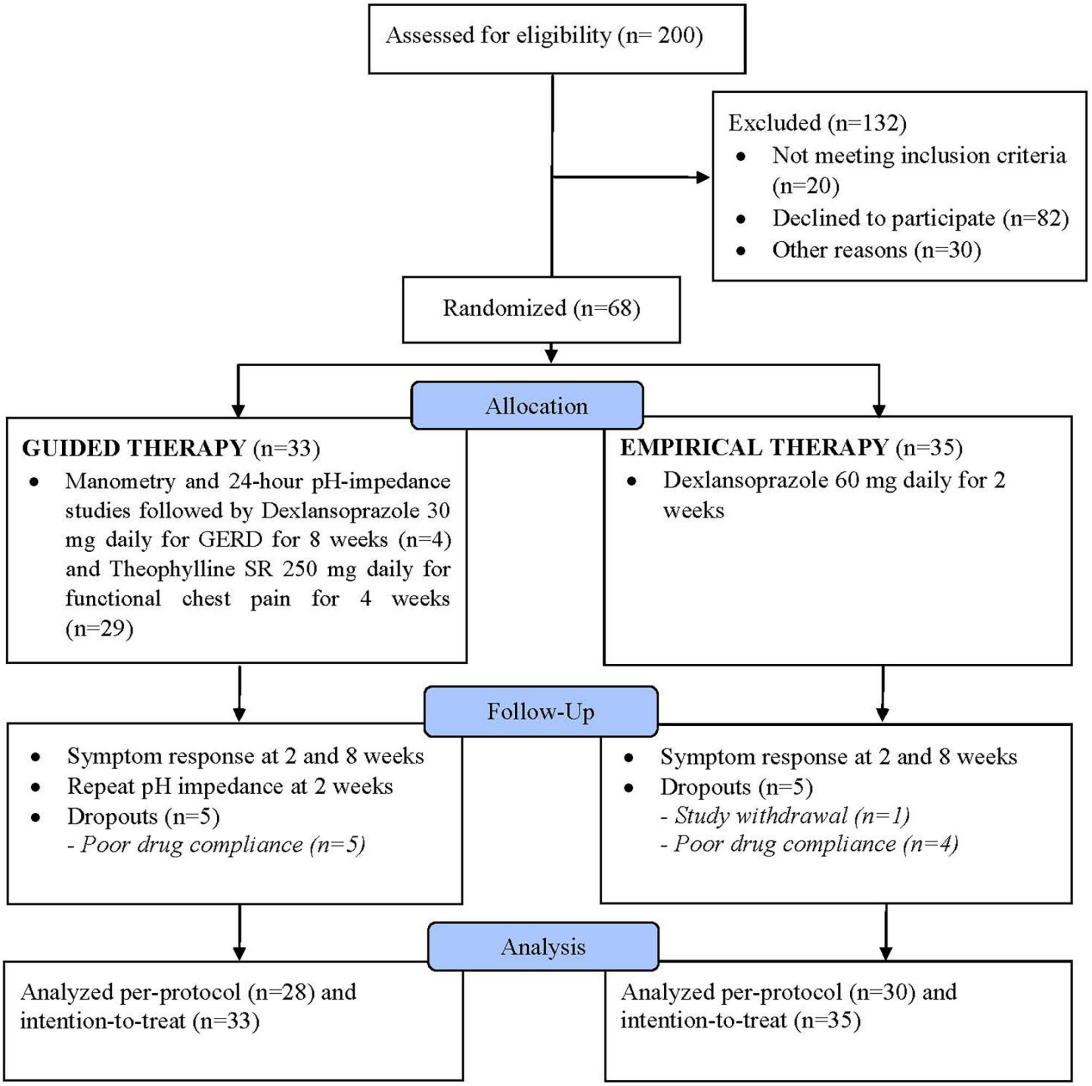
Flow chart of the study.

Of 200 screened patients, 68 were eventually enrolled and 132 excluded with a decline to participate being the main reason in 82/132 patients (Figure 1). Of the 68 enrolled participants, 33 were randomized into the guided therapy group and 35 into the empirical therapy group. Overall, the baseline characteristics were similar between both groups. There was no difference in the mean age of participants in the guided and empirical groups (46.5 ± 15.1 and 47.6 ± 12.3 years, respectively) (*P* = 0.7). Participants were predominantly males (guided 63.6% vs. empirical 51.4%) (*P* = 0.4). Although no difference in the mean BMI between groups, participants were considered overweight (guided 27.4 ± 4.3 vs. empirical 27.1 ± 7.2 kg/m^2^) (*P* = 0.8). In addition, both groups had similar QOLRAD (guided 21.3 vs. empirical 21.7, *P* = 0.8) and GERDQ (guided 8.4 vs. empirical 8.7, *P* = 0.7) scores.

By the end of the trial, there were five (5) participants from each group who were “drop-outs” either they withdrew from the study or because of poor compliance or unable to tolerate the prescribed medications. After an 8-week follow-up, there were 28 participants from the guided group and 30 participants from the empirical group who had completed the whole study (i.e., per-protocol) (Figure 1).

Erosive esophagitis was present in 19.1% of all participants (*n* = 13/68). In the guided group, based on the pH-impedance test, 12.1% had GERD (*n* = 4/33), 33.3% with functional chest pain (*n* = 11/33), and 54.5% with reflux hypersensitivity (*n* = 18/33). In the guided therapy group, four participants were given dexlansoprazole 30 mg daily for GERD for 8 weeks and the remaining 29 participants were given theophylline SR 250 mg daily for 4 weeks. On the other hand, in the empirical group, of participants consented to the pH-impedance test, none had GERD, 47.1% had functional chest pain (*n* = 8/17), and 52.9% with reflux hypersensitivity (*n* = 9/17). All participants in the empirical group were given dexlansoprazole 60 mg daily for 2 weeks, regardless of the pH-impedance findings.

### Primary Outcome

**Figure 2:**
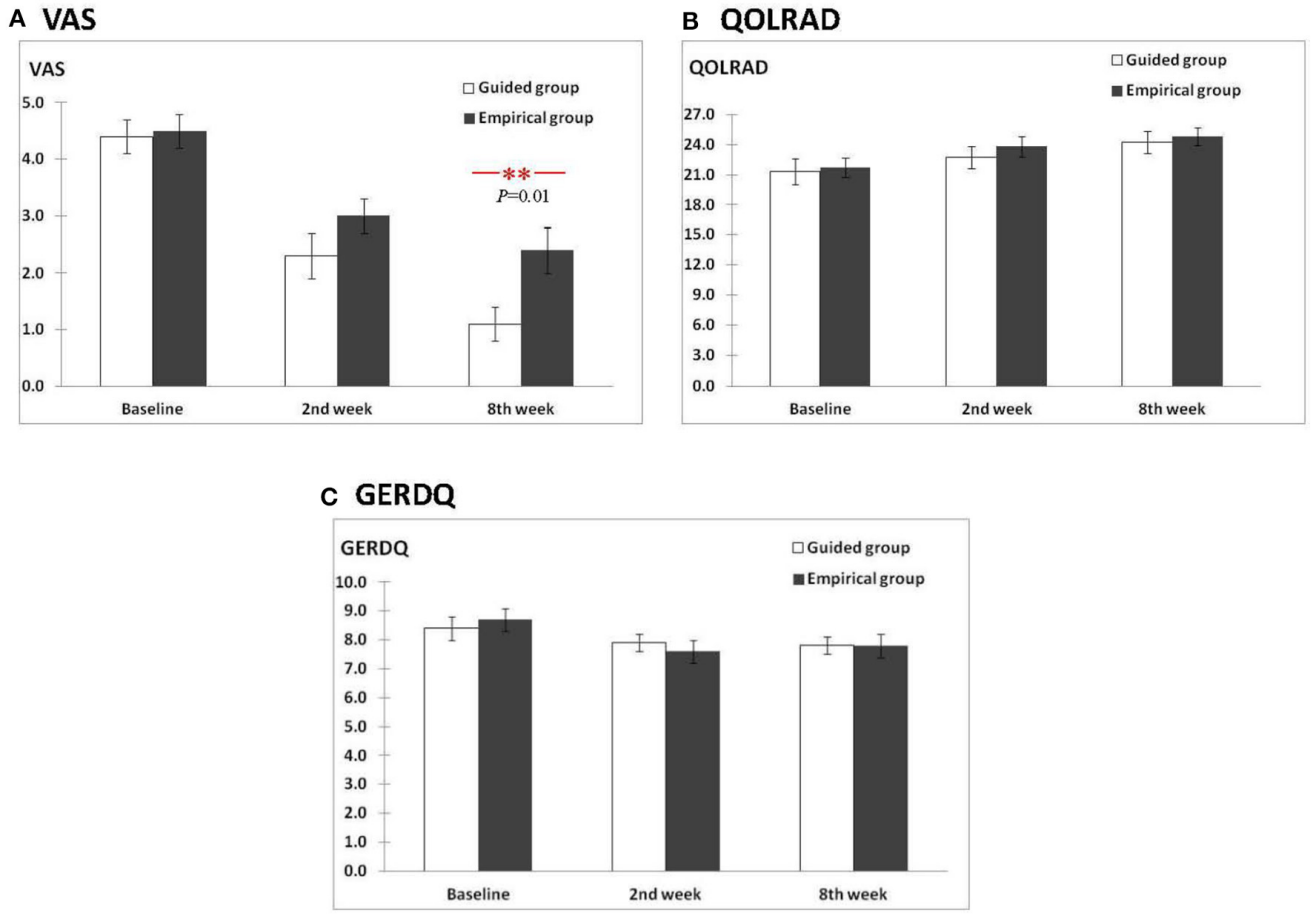
Comparison of scores between the guided and empirical groups for **(A)** VAS, **(B)** QOLRAD, and **(C)** GERDQ.

Per-protocol, there were no between-group differences in the mean chest pain VAS at baseline and 2nd week (all *P* > 0.05) (Figure 2A). However, in the 8th week, significantly lower mean VAS was reported with guided vs. empirical groups (1.0 vs. 2.6, *P* = 0.005). For within-group differences, using the ANOVA test, the mean chest pain VAS scores differed significantly between baseline, 2nd, and 8th weeks for both groups (both *P* < 0.01) (Table 1). In the guided group, the mean VAS score was significantly lower in the 8th week from baseline (*P* ≤ 0.001). In the empirical group, the mean VAS score was significantly lower at 2nd week from baseline (*P* = 0.007) and at 8th week from baseline (*P* = 0.009), but no difference was observed between 2nd and 8th weeks (*P* = 0.8). Similar results were reported with the intention-to-treat approach (Supplementary Figure 1).

**Table 1:** Results of within-group differences between variable scores of the guided and empirical groups.

	**Guided group (*****n*** **=** **28)**		**Empirical group (*****n*** **=** **30)**	
	**MD (95% CI)**	***P*-value**	**MD (95% CI)**	***P*-value**
**VAS**				
Baseline−2nd week	2.4 (1.5, 3.2)	**<0.001**	1.4 (0.3, 2.5)	**0.007**
Baseline−8th week	3.5 (2.7, 4.4)	**<0.001**	1.8 (0.4, 3.3)	**0.009**
2nd week−8th week	1.2 (0.4, 1.9)	**0.001**	0.4 (-0.5, 1.3)	0.751
**QOLRAD**				
Baseline−2nd week	−1.1 (−2.7, 0.5)	0.257	−2.4 (−4.4, −0.5)	**0.011**
Baseline−8th week	−3.0 (−5.3, −0.8)	**0.006**	−2.6 (−5.3, 0.1)	0.058
2nd week−8th week	−1.9 (−3.9, 0.1)	0.066	−0.2 (−2.4, 1.9)	1.000
**GERDQ**				
Baseline−2nd week	0.2 (1.0, 1.4)	1.000	1.2 (−0.2, 2.6)	0.135
Baseline−8th week	0.5 (−0.7, 1.7)	0.987	1.0 (−0.1, 2.1)	0.097
2nd week−8th week	0.3 (−0.8, 1.3)	1.000	−0.2 (−1.3, 0.9)	1.000
**pH PARAMETERS**				
**(Baseline−2nd week)**				
% time acid exposure	0.3 (−0.5, 1.1)	0.455	–	–
DeMeester score	0.7 (−1.9, 3.3)	0.563	–	–

*MD, mean difference. Bold values are used to indicate statistical significance*.

### Secondary Outcomes

Per-protocol, for the mean QOLRAD scores, no between-group differences were observed for guided vs. empirical at baseline, 2nd, and 8th weeks (all *P* > 0.05) (Figure 2B). For within-group differences using the ANOVA test, the mean QOLRAD scores did not differ significantly between baseline, 2nd, and 8th weeks for both groups (i.e,. all *P* > 0.05 for guided and empirical, respectively). Using the *t*-test, in the guided group, the mean QOLRAD score increased significantly from baseline to 8th week (*P* = 0.006), but not from baseline to 2nd week (*P* = 0.3) and from 2nd week to 8th week (*P* = 0.1) (Table 1). Likewise, using the *t*-test, in the empirical group, the mean QOLRAD score increased earliest at 2nd week from baseline (*P* = 0.01) but not sustained beyond 2nd week (Table 1). However, with the intention-to-treat approach, improvement in QOLRAD was also seen in the 8th week from baseline (*P* = 0.01) (Supplementary Table 1).

On the other hand, no between-group differences in the mean GERDQ scores were observed at baseline, 2nd, and 8th weeks (all *P* > 0.05) (Figure 2C). For within-group differences, regardless of the ANOVA or *t*-test, there were no within-group differences in the mean GERDQ scores between baseline, 2nd and 8th weeks for both groups (all *P* > 0.05) (Table 1).

For the pH parameters, data for within-group analysis was only available for 22 participants in the guided group (Table 1). There were no significant differences in the % time of acid exposure and the DeMeester score after intervention in the guided group (both *P* > 0.05).

### Safety and Adverse Events

The interventions were relatively safe with none of the study participants experienced any fatal adverse events. However, five participants experienced mild to moderate adverse events, including two (*n* = 2) from the guided group who reported moderate palpitations, two (*n* = 2) from the empirical group who experienced mild abdominal pain, and one (*n* = 1) from the empirical group who reported mild maculopapular rashes.

## Discussion

The following is a summary of our findings (1) guided therapy was effective over empirical therapy in reducing global chest pain scores at 8th week but not earlier, (2) no difference was observed in QOLRAD between groups but for guided therapy, QOLRAD was better beginning only at 8th week. Instead, improvement of QOLRAD with empirical therapy was earlier at 2nd week, and lastly (3) therapies were safe with only a few reports of adverse events.

GERD-related disorders affect ~60% of patients with non-cardiac chest pain (3, 19, 20). In the real-life practice where pH-impedance tests are not commonly available, most physicians would have started their patients with empirical PPI therapy but it is unknown if such an approach is less effective than phenotyping patients first by using the pH or pH-impedance test. The current study indicates that guided therapy i.e., phenotype first and then treat based on phenotype was likely the more effective approach than empirical therapy when it comes to global chest pain relief at a longer-term. A limitation of this study is that the empirical PPI arm did not continue until 8 weeks unlike the guided arm but in actual clinical practice and since ours is a pragmatic clinical trial, PPI given for 2 weeks is the typical duration for empirical therapy.

In our study, dexlansoprazole (Dexilant®) was the chosen PPI, a novel dual delayed delivery of lansoprazole which was approved by the U. S. Food and Drug Administration (FDA) for treating erosive and non-erosive reflux-related heartburn (21). Studies using dexlansoprazole as an empirical agent (PPI test) or for the indication of acid-related chest pain are limited (22, 23), however, our study indicates that this agent was effective for chest pain but also safe with additional advantages of single dosing and not meal dependent due to its unique properties. While many considered PPI especially lansoprazole as relatively safe however minor side-effects may have been more common in actual practice than not. It was observed that within the empirical group, one participant reported mild abdominal discomfort and the other participant complained of mild maculopapular rashes.

A previous study has observed that reflux-related chest pain significantly affected QOLRAD including poor physical and mental functioning (24). We reported similar findings with a lower overall QOLRAD at baseline for both groups. However, our trial did not find a difference between treatment groups indicating that both approaches did improve QOLRAD similarly but we cannot exclude the possibility of placebo effects. Within the treatment group, improvement in QOLRAD was seen earlier at 2nd week with empirical therapy; however, for guided therapy, QOLRAD only improved at 8th week and not earlier. This might be explained partly due to intolerance from theophylline that had occurred at the onset of therapy in the guided group.

In our study, theophylline SR, a xanthine derivative known for its bronchodilation property, was our chosen pain modulator. Although an “old” drug, the choice was largely pragmatic since this agent is readily available in our hospital formulary, the cost is cheap but most importantly in previous trials, theophylline has demonstrated substantial clinical benefits in patients with chest pain due to hypersensitive esophagus (25–27). Other agents including tricyclic antidepressant, SSRI, or SNRI were less tolerable, more expensive, and less often prescribed in our setting. Furthermore, our studies have excluded those with psychological co-morbids where these agents were more effective. As with previous studies, the efficacy of theophylline was replicated in our trial but in contrast, we used the sustained release formulation, given as a single dose, and therefore better tolerated with less adverse effects. While theophylline is known for its side-effects including palpitation and nausea however in our study these adverse effects were relatively mild and few with only two participants reported palpitations. The actual mechanism of how theophylline works in reflux hypersensitivity is unclear but its actions on the adenosine receptor may be important. Recent animal studies indicate that sustained activation of adenosine A_2B_ receptors on myeloid cells could transactivate nociceptors of sensory neurons (28), and that systemic administration of A_2B_ antagonists (MRS-1754 and PSB-1115) could reduce pain in response to stress in irritable bowel syndrome (29).

Other secondary outcomes have included GERDQ scores and pH parameters (% time of acid exposure and DeMeester score). These outcomes were not significantly different between intervention groups but also within groups. The results are probably not that surprising since participants with true GERD were small in number, and that treatment with dexlansoprazole was highly effective in both groups thus canceling out any therapeutic effects.

There are a number of limitations to the current study. The sample size was relatively small due to the high screen failure rate and because of trial methodology but the groups were well-matched for age and sex at baseline. However, the phenotypes of GERD were unequal in both groups with a large majority being functional or reflux hypersensitivity. This might be due to our study setting where most referred patients with chest pain were likely of the refractory GERD phenotype. Other limitations included unequal treatment duration of empirical and guided groups and no further follow-ups including the measurement of pH impedence tests beyond 8 weeks. Since we designed the study as a pragmatic trial, therefore the unequal duration of different treatment options was unavoidable. In addition, the choice of theophylline, an old drug rather than newer neuromodulators could be debated and might not seem to be the best choice based on current setting. On that note, we have described in detail in previous section the reasons for our choice on theophylline, and although an old agent, many practitioners in our setting have found theophylline SR somehow effective, more tolerable and has less stigma attached to “anti-depressant.” Lastly, since both treatment groups had a similar number of drop-outs at the end, any differences in results between the two forms of approach i.e., per-protocol and intention-to-treat would have been canceled out, and this was what we have observed in our analysis. Although a pragmatic trial, per-protocol results were probably preferable to assess longer-term therapeutic effects.

## Conclusion

In this pragmatic trial, the guided approach may be preferred over empirical therapy in terms of global chest pain response especially if most patients have the functional or hypersensitivity phenotype. QOL and other acid-related symptoms or parameters do not seem to differ between the two groups. Therapies including dexlansoprazole and theophylline SR given in both approaches are safe but longer-term follow-up is needed.

## Data Availability Statement

The raw data supporting the conclusions of this article will be made available by the corresponding author, upon reasonable request.

## Ethics Statement

The studies involving human participants were reviewed and approved by Human Research Ethics Committee, USM (reference: USM/JEPeM/14070265). The patients/participants provided their written informed consent to participate in this study.

## Author Contributions

NK, MA, CA, and YL participated in study design. NK, MA, CA, TJ, SN, AA, ZY, HL, and YL were responsible for study recruitment and data acquisition. NK, ZM, NH, and YL conducted the statistical analysis and interpreted the data. NK, ZM, and YL revised the manuscript. All authors approved the final version of the article, including the authorship list.

## Conflict of Interest

The authors declare that the research was conducted in the absence of any commercial or financial relationships that could be construed as a potential conflict of interest.

## Abbreviations

GERD: Gastroesophageal reflux disease
GERDQ: GERD questionnaire
LES: The lower esophageal sphincter
PPI: Proton-pump inhibitor
QOL: Quality of life
QOLRAD: Quality of Life in Reflux and Dyspepsia
VAS: Pain visual analog score.
