# Supplementary material for: Comparing Efficacy and Safety of Empirical vs. Guided Therapy for Non-cardiac Chest Pain: A Pragmatic Randomized Trial
Source: Front Med (Lausanne). 2021 Feb 15;8:605647. doi: 10.3389/fmed.2021.605647 (PMC7917139; doi:10.3389/fmed.2021.605647)
Supplement: Supplementary file 1 [file Table_1.docx]

**Supplementary Table 1. Results of within-group differences between variable scores of the guided and empirical groups.**

|  | **Guided group (n=33)** | |  | **Empirical group (n=35)** | |
| --- | --- | --- | --- | --- | --- |
|  | **MD (95% CI)** | **P-value** |  | **MD (95% CI)** | **P-value** |
|  |  |  |  |  |  |
| **VAS** |  |  |  |  |  |
| Baseline - 2^nd^ week | 2.1 (1.3, 3.0) | **<0.001** |  | 1.5 (0.5, 2.5) | **0.001** |
| Baseline - 8^th^ week | 3.3 (2.5, 4.1) | **<0.001** |  | 2.0 (0.7, 3.3) | **0.001** |
| 2^nd^ week - 8^th^ week | 1.2 (0.5, 1.8) | **0.001** |  | 0.5 (-0.2, 1.3) | 0.286 |
|  |  |  |  |  |  |
| **QOLRAD** |  |  |  |  |  |
| Baseline - 2^nd^ week | -1.4 (-3.1, 0.2) | 0.096 |  | -2.1 (-4.0, -0.3) | **0.020** |
| Baseline - 8^th^ week | -3.0 (-5.2, -0.8) | **0.006** |  | -3.1 (-5.6, -0.5) | **0.013** |
| 2^nd^ week - 8^th^ week | -1.5 (-3.4, 0.4) | 0.149 |  | -1.0 (-3.1, 1.2) | 0.823 |
|  |  |  |  |  |  |
| **GERDQ** |  |  |  |  |  |
| Baseline - 2^nd^ week | 0.6 (-0.6, 1.7) | 0.689 |  | 1.1 (-0.1, 2.3) | 0.091 |
| Baseline - 8^th^ week | 0.7 (-0.4, 1.8) | 0.407 |  | 0.9 (-0.1, 1.8) | 0.099 |
| 2^nd^ week - 8^th^ week | 0.1 (-0.8, 1.1) | 1.000 |  | -0.2 (-1.1, 0.7) | 1.000 |

MD,mean difference.
