# Supplementary material for: Comparing Efficacy and Safety of Empirical vs. Guided Therapy for Non-cardiac Chest Pain: A Pragmatic Randomized Trial
Source: Front Med (Lausanne). 2021 Feb 15;8:605647. doi: 10.3389/fmed.2021.605647 (PMC7917139; doi:10.3389/fmed.2021.605647)

Supplementary Figure 1. Comparison of scores between the guided and empirical groups for (a) VAS, (b) QOLRAD, and (c) GERDQ using intention-to-treat approach.

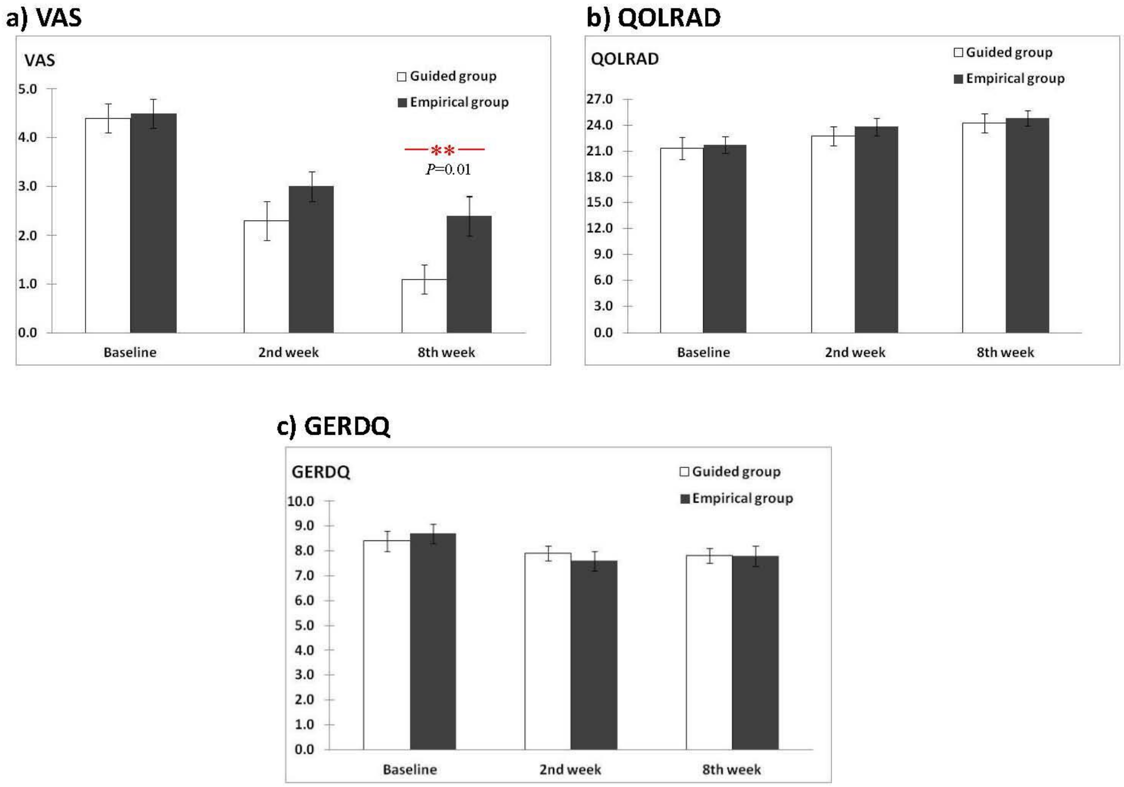

Supplement: Supplementary file 2 [file Image_1.pdf]
